# Salivary levels of azurocidin and soluble azurophilic granules in periodontal disease

**DOI:** 10.25122/jml-2023-0286

**Published:** 2024-02

**Authors:** Fatima Sabah Jasim, Batool Hassan Al-Ghurabi, Lubaba Abdulsamad Abdulameer

**Affiliations:** 1 Department of Basic Science of Oral Microbiology, College of Dentistry, University of Baghdad, Baghdad, Iraq; 2 Department of Basic Science, College of Dentistry, University of Baghdad, Baghdad, Iraq; 3 Department of Periodontics, College of Dentistry, University of Baghdad, Baghdad, Iraq

**Keywords:** periodontal disease, neutrophils, azurocidin, azurophilic granule, CD63

## Abstract

Periodontitis is an infection-driven inflammatory condition of the periodontium. Neutrophils are one of the most important first-line immune cells that protect against pathogen microorganisms in the saliva, but they may also mediate tissue death in inflammatory disorders. The aim of our study was to estimate salivary levels of azurocidin and extracellular azurophilic granules cluster of differentiation (CD63) as biomarkers of neutrophil activation in patients with periodontal diseases and to study the correlation between the levels of these two biomarkers and clinical periodontal parameters. The study included 60 patients with periodontal disease (30 patients with periodontitis and 30 with gingivitis) and 25 healthy controls. The assessed parameters were bleeding on probing, the plaque index, clinical attachment loss, and probing pocket depth. Saliva samples were taken from each study participant, and azurocidin and CD63 levels were measured using ELISA. Azurocidin and CD63 levels were significantly higher in patients with periodontitis and patients with gingivitis than in controls (*P* < 0.05), and significantly higher in patients with periodontitis than in patients with gingivitis (*P* < 0.05). Moreover, we found a significant positive correlation between the two biomarkers with clinical attachment loss in the periodontitis group. This study has shown that increased salivary azurocidin and extracellular CD63 levels are associated with enhanced innate response in periodontal disease and can be considered biomarkers of neutrophil activation.

## INTRODUCTION

Periodontal diseases affect the periodontium, the structure that supports the tooth and is made up of the alveolar bone, gingiva, cementum, and periodontal ligament. The most prevalent periodontal disease, gingivitis, affects up to 90% of individuals; however, improved oral hygiene can help reverse this reactive condition [1]. Periodontitis develops when gingivitis progresses into a chronic, serious, and irreversible inflammatory condition. Bacteria may then spread further into the periodontium and tissues. However, host defenses against the invasive microorganisms may lead to the loss of the periodontium’s attachment to the alveolar bone and increase the risk of tooth loss [2,3].

Neutrophils are one of the first inflammatory cells to migrate to the site of inflammation through chemotaxis [4]. Azurocidin (AZU), often referred to as cationic antimicrobial protein 37 kDa (CAP37) or heparin-binding protein (HBP), is an inactive homolog of serine proteinases found in granulocytes and an important mediator of inflammatory response [5,6]. Tetraspanins are a large superfamily of cell surface membrane proteins identified by four transmembrane domains. They are involved in various biological processes, including tumor invasion, adhesion, differentiation, and cell activation. Tetraspanin-enriched microdomains, or protein networks made by tetraspanins, are found at the cell surface. The first tetraspanin discovered was cluster of differentiation 63 (CD63), a membrane protein found primarily in azurophilic granules that control the quality of antimicrobial protein, also found in late endosomes and lysosomes. At the cell surface, CD63 is endocytosed by a clathrin-dependent process [7].

The aim of this study was to quantify salivary levels of AZU and CD63 as biomarkers of neutrophil activation in patients with periodontal disorders and investigate the relationship between these two biomarkers and clinical periodontal parameters.

## PATIENTS AND METHODS

### Study design

We conducted a case–control study from November 2022 to January 2023. The study included 85 participants separated into two groups: 1) a study group of 60 patients (30 patients with periodontitis and 30 patients with gingivitis); 2) a control group of 25 age- and gender-matched individuals with clinically healthy periodontium. All study participants were recruited from the clinic of the Department of Periodontics, College of Dentistry, University of Baghdad. A specialist dentist performed an oral examination for every participant. The periodontal status of all teeth was assessed using a Williams graduated periodontal probe; periodontal parameters included the plaque index (PLI), bleeding on probing (BOP), probing pocket depth (PPD), and clinical attachment loss (CAL).

### Inclusion and exclusion criteria

We included patients who were systemically healthy, were not smoking or drinking heavily, had no periodontal treatment in the previous 6 months, were not frequently using medications, and had at least 20 or more natural teeth. The gingivitis group included patients with generalized gingivitis with intact periodontium, in whom >30% of sites were affected with BOP, with no PPD > 3 mm and no CAL [8]. The periodontitis group included patients with interdental CAL > 2 mm for non-adjacent teeth or buccal/oral CAL ≥ 3 mm with pockets of >3 mm at more than two teeth. In patients with generalized periodontitis the bone loss exceeded 30% of teeth, with PPD > 5 mm, or PPD > 4 mm with BOP. Patients in the control group had healthy intact periodontium, BOP < 10%, PPD ≤ 3 mm, and no CAL. We excluded patients who were pregnant, were receiving orthodontic treatment, or who had undergone previous periodontal therapy.

### Sample collection

After the oral examination, the participants were asked to wash their mouths thoroughly with water to remove any debris or contaminating material. During sample collection, the participants were asked to sit in a relaxed position and avoid movements of the tongue, jaw, lips and cheeks. With the lips slightly open, the participants let the saliva drool passively over the lower lip into the test tube, without spitting or swallowing, as described in the literature [9]. Blood-contaminated samples were discarded. The participant’s identification number, which was previously recorded on the case sheet, was inscribed on the tube’s label. The samples were centrifuged at 2,500 rpm for 20 min to separate the clear supernatant, which was then aspirated into Eppendorf tubes and placed in a freezer at −80 °C until the day of the analysis.

### Determination of salivary biomarkers

All participants underwent an assessment to measure the salivary levels of AZU and CD63 biomarkers (MyBioSource) using an automated ELISA system.

### Statistical analysis

We used SPSS 21 (IBM Corp) for data description, analysis, and presentation. The normality of quantitative variable distributions across groups was assessed with the Shapiro–Wilk test. Differences in mean rank between independent groups were assessed using the Kruskal–Wallis test with the Dunn–Bonferroni approach. When the predicted cell counts were less than 5, the distribution relationship between two qualitative variables was examined using the chi-squared test. The monotonic correlation between two variables that were not regularly distributed was determined using the Spearman correlation test. Additional intergroup comparisons were undertaken in cases of significance using the Bonferroni post hoc test, and a *P* value of <0.05 was deemed statistically significant.

## RESULTS

The values of clinical periodontal parameters in patients and controls are presented in Table 1. We found a significant elevation in the mean rank value of AZU among patients with periodontitis and gingivitis compared to the control group (*P* < 0.05; Table 2). After using multiple pairwise comparisons, we found significant differences between each group (*P* < 0.05). Furthermore, we found a significant positive correlation between the level of AZU and CAL in the periodontitis group but no significant correlations between AZU and periodontal parameters in the gingivitis group (Table 3).

**Table 1:** Median values of clinical parameters in the two groups

	Groups	Median	Mean rank	Kruskal–Wallis	*P* value	Dunn–Bonferroni MPC	*P* value
PLI	Periodontitis	45.80	54.55	43.177	<0.0001	Periodontitis–gingivitis	0.999
	Gingivitis	49.00	54.15			Periodontitis–control	<0.0001
	Control	12.00	15.76			Gingivitis–control	<0.0001
BOP	Periodontitis	28.79	50.35	53.110	<0.0001	Periodontitis–gingivitis	0.361
	Gingivitis	35.00	60.25			Periodontitis–control	<0.0001
	Control	6.00	13.48			Gingivitis–control	<0.0001
PPD	Periodontitis	4.40					
CAL	Periodontitis	4.00					

MPC, multiple pairwise comparisons

**Table 2:** Salivary level of AZU (ng/ml) in the two groups

Groups	Median	Mean rank	Kruskal–Wallis	*P* value	Dunn–Bonferroni MPC	*P* value
Periodontitis	178.188	63.67	58.001	<0.0001	Periodontitis–gingivitis	0.028
Gingivitis	156.510	47.07			Periodontitis–control	<0.0001
Control	66.614	13.32			Gingivitis–control	<0.0001

**Table 3:** Correlation between AZU and periodontal parameters

Groups	PLI		BOP		PPD		CAL	
	R	*P* value	R	*P* value	R	*P* value	R	*P* value
Periodontitis	0.268	0.153	0.274	0.143	0.137	0.469	0.607	<0.0001
Gingivitis	0.018	0.926	−0.241	0.199				

The mean rank values of extracellular CD63 for the study and control groups are shown in Table 4. There were significant differences between groups; the periodontitis group had the highest mean value among the three groups, with 69.27 pg/ml, followed by the gingivitis group with a mean value of 39.67 pg/ml, and the control group with a mean value of 15.48 pg/ml (*P* < 0.05). In intergroup comparisons of CD63 levels across all pairs of groups, there was a substantial difference between each group and the others, but there was only one significant correlation, between CD63 and CAL in the periodontitis group (Table 5).

**Table 4:** Salivary level of CD63 (pg/ml) in the two groups

Groups	Median	Mean rank	Kruskal–Wallis	*P* value	Dunn–Bonferroni MPC	*P* value
Periodontitis	11.377	69.27	65.608	<0.0001	Periodontitis–gingivitis	<0.0001
Gingivitis	7.263	39.67			Periodontitis–control	<0.0001
Control	4.646	15.48			Gingivitis–control	0.001

**Table 5:** Correlation between CD63 and periodontal parameters

Groups	PLI		BOP		PPD		CAL	
	R	*P* value	R	*P* value	R	*P* value	R	*P* value
Periodontitis	0.059	0.756	−0.010	0.956	0.301	0.106	0.437	0.016
Gingivitis	−0.043	0.822	−0.343	0.064				

## DISCUSSION

The findings of this study demonstrated that high salivary levels of AZU were significantly associated with periodontal disease, as all patients with periodontitis and gingivitis had increased AZU levels compared to controls. Moreover, there were significant differences between the periodontitis and gingivitis groups, suggesting that the elevation of AZU levels might be involved in the progression of periodontal disease. Increased neutrophil infiltration and AZU release in inflammatory periodontal tissues may cause elevated salivary AZU levels in patients with periodontal disease. In addition, AZU has a high affinity for the lipopolysaccharides found in the cell wall of Gram-negative bacteria, the primary cause of periodontal disease [10–12]. As a result, individuals with periodontitis have increased salivary levels of AZU, as previously reported in the literature [13–17]. We found no significant correlation between AZU and clinical periodontal parameters, except for CAL in the periodontitis group; similar results were obtained by a study that demonstrated a significant correlation between AZU and all periodontal parameters, including CAL in patients with periodontitis [13]. However, there is sufficient evidence to show that a sizeable amount of inflammatory damage to periodontal tissues is caused by neutrophils that are either overactive or overly numerous and cause collateral damage [18]. They can destroy pathogens through phagocytosis, intracellular death via oxidative and proteolytic processes, inflammatory responses, and extracellular mechanisms such as degranulation and the release of mediators produced by neutrophil granules [18–20].

We found a significant elevation in salivary levels of CD63 in the periodontitis and gingivitis groups compared to healthy controls, and a significant increase in CD63 levels in the periodontitis group compared to the gingivitis group. In contrast, another study that examined the concentration of CD63 in GCF and saliva by ELISA did not find significant differences between the three groups in the saliva samples [21]. In the same study, gingival crevicular fluid levels were lower in patients with gingivitis compared to patients with periodontitis.

Extracellular vehicles (EVs) have been involved in the pathogenesis of several chronic inflammatory, autoimmune, and infectious diseases. They can modify and activate target cells through paracrine or endocrine pathways, mediating immune responses between distant cells [22,23]. Due to their abundance in several bodily fluids, such as plasma, urine, saliva, and breast milk, EVs are now considered possible diagnostic and therapeutic tools for several disorders [24–26]. A recent study revealed that individuals with periodontal disease have higher overall EV concentrations [21].

Our analysis has shown that CAL and salivary CD63 levels were correlated in patients with periodontitis. This finding is in contrast with those reported by other studies, which did not find meaningful relationships between CD63 levels in the saliva and any of the clinical periodontal factors [21,27]. This study also revealed a positive correlation between AZU and CD63 in patients with gingivitis, confirming that high levels of these biomarkers may reflect an increase in an innate immune response and activation of neutrophils in patients with periodontal diseases.

### Study limitations

The present study has some limitations. We did not take into consideration the different stages and grades of periodontitis, and did not perform a post-treatment evaluation to assess the relationship between the improvement of clinical parameters and the level of neutrophil mediators. Furthermore, soluble markers are only surrogate markers of leukocyte action and may not fully reflect their activation status.

## CONCLUSION

The results of this study suggest that increased salivary AZU and CD63 levels could be associated with enhanced innate response in periodontal disease and serve as biomarkers of neutrophil activation.
